# Tuning Polymer–Metal Interfaces via Solvent-Engineered Electroless Nickel Coatings on Functional Fibres

**DOI:** 10.3390/polym17121693

**Published:** 2025-06-18

**Authors:** Chenyao Wang, Heng Zhai, Xuzhao Liu, David Lewis, Yuhao Huang, Ling Ai, Xinyi Guan, Hugh Gong, Xuqing Liu, Anura Fernando

**Affiliations:** 1 Department of Materials, The University of Manchester, Manchester M13 9PL, UK; chenyao.wang-3@postgrad.manchester.ac.uk (C.W.); xuzhao.liu@manchester.ac.uk (X.L.); yuhao759@gmail.com (Y.H.); x.guan@hud.ac.uk (X.G.); hugh.gong@manchester.ac.uk (H.G.); xqliu@nwpu.edu.cn (X.L.); 2 Department of Chemical Engineering, The University of Manchester, Manchester M13 9PL, UK; david.lewis-4@manchester.ac.uk (D.L.); ling.ai@manchester.ac.uk (L.A.); 3 Technical Textile Research Centre, University of Huddersfield, Huddersfield HD1 3DH, UK

**Keywords:** polymerisation treatment, surface activation, solvent-assisted electroless deposition, nanocrystalline coatings, conductive functional textile, thermal stability

## Abstract

Electroless nickel deposition (ELD) on polymer substrates enables the fabrication of flexible, conductive fibres for wearable and functional textiles. However, achieving uniform, low-defect coatings on synthetic fibres such as nylon-6,6 remains challenging due to their chemical inertness, hydrophobicity, and poor interfacial compatibility with metal coatings. This study presents a solvent-assisted approach using dimethyl sulfoxide (DMSO) in a conventional aqueous ELD bath to control both polymer–metal interfacial chemistry and nickel coating microstructure. The modified surface supports dense catalytic sites, triggering spatially uniform Ni nucleation. The combination of scanning electron microscopy and transmission electron microscopy confirms the difference in coarse grains with fully aqueous baths to a nanocrystalline shell with DMSO-modified baths. This refined microstructure relieves residual stress and anchors firmly to the swollen polymer, delivering +7 °C higher onset decomposition temperature and 45% lower creep strain at 50 °C compared with aqueous controls. The fabric strain sensor fabricated by 1 wt.% DMSO-modified ELD shows a remarkable sensitivity against strain, demonstrating a 1400% resistance change under 200% stain. Electrochemical impedance and polarisation tests confirm a two-fold rise in charge transfer resistance and negligible corrosion current drift after accelerated ageing. By clarifying how a polar aprotic co-solvent couples polymer swelling with metal growth kinetics, the study introduces a scalable strategy for tuning polymer–metal interfaces and advances solvent-assisted ELD as a route to mechanically robust, thermally stable, and corrosion-resistant conductive textiles.

## 1. Introduction

Lightweight aliphatic polyamides, most prominently nylon-6,6, have become indispensable in wearable electronics, electromagnetic-shielding fabrics, and soft-robotic skins because they unite high toughness, elasticity, and textile processability [1,2,3]. Translating those attributes into functional devices, however, demands robust electrical pathways on the polymer surface. It is challenging that the low surface energy of nylon-6,6 and its pronounced modulus mismatch with metals make polymer–metal hybrids vulnerable to microcracking, delamination, and catastrophic loss of conductivity under thermal or mechanical load [4]. Electroless deposition (ELD) is a widely adopted technique for metallising non-conductive substrates, such as polymers, ceramics, and textiles, through an autocatalytic redox reaction between a reducing agent and nickel ions in solution [5,6]. Among textile substrates, nylon-6,6 yarns have garnered particular attention due to their flexibility, low weight, and broad applicability in wearable electronics, electromagnetic shielding, flexible sensors, and other functional textile systems [7]. However, fully aqueous ELD baths often exhibit rapid deposition kinetics that can lead to excessive coating thickness, internal stress accumulation, and compromised thermal and mechanical properties [8]. These limitations are especially problematic in soft, deformable substrates like nylon-6,6 yarns, which must maintain performance under high strain or repeated flexing. A particular challenge arises from the inherent material mismatch: a hard, brittle metal layer deposited on a soft, elastic polymer fibre often leads to microcracking, delamination, or poor adhesion, especially under thermal or mechanical load. These interfacial failures can reduce coating conductivity, sensor reliability, and long-term durability, critical drawbacks in applications such as wearable electronics or flexible circuits.

From a polymer-science perspective, two additional factors are critical for achieving reliable metallisation. First, the polymer surface must be functionalised with chemical groups that can simultaneously interact strongly with the polymer substrate and effectively bind metal ions. Bio-inspired polydopamine (PDA) self-assembles through oxidation-induced polymerisation, while polyphenolic tannic acid (TA) supplies complementary catechol and galloyl moieties. The resulting catechol-rich layer presents abundant hydroxyl (-OH) and amine (-NH) groups capable of forming hydrogen bonds with nylon-6,6 amide linkages and chelating Ni^2+^ ions, thereby strengthening the polymer–metal interface [9,10,11]. Second, solvent-assisted control of chain mobility can drastically alter interphase chemistry. Polar aprotic solvents such as dimethyl sulfoxide (DMSO) transiently disrupt inter-chain hydrogen bonding in polyamides, increasing free volume and swelling amorphous domains. When DMSO is introduced directly into an ELD bath, it is poised to plasticise the nylon-6,6 surface, enhancing the infiltration of catalyst ions and PDA/TA oligomers, and modulate nickel ion solvation, slowing reduction kinetics and encouraging finer, low-stress grain growth.

**Figure 1:**
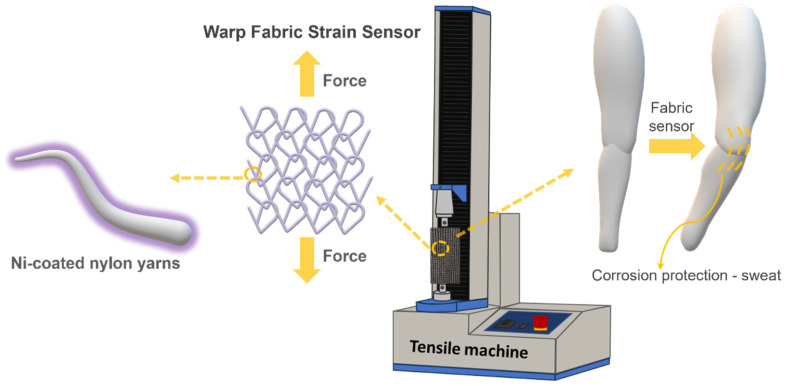
Illustration of a wearable application using DMSO-modified nickel-coated polymer fibres integrated into a strain-sensing knitted fabric.

Despite these polymer-centric advantages, the direct incorporation of DMSO as a co-solvent in electroless nickel plating of textile fibres has not yet been explored. Previous attempts to address the challenges associated with fully aqueous electroless nickel deposition, such as excessive coating thickness, internal stress accumulation, and compromised thermal and mechanical properties, have primarily focused on modifying aqueous bath chemistry through adjustments in pH, the introduction of complexing agents, or employing organic solvents as pretreatment solutions to sensitise polymer surfaces before metal deposition [12,13]. While these approaches can mitigate some deposition challenges, the direct incorporation of non-aqueous co-solvents into the ELD bath itself, particularly polar aprotic solvents, remains largely unexplored. Polar aprotic solvents exhibit unique abilities to alter ion mobility, metal–ion solvation, and substrate interactions differently than water, potentially enabling slower, more uniform metal deposition [14,15,16]. Among these solvents, DMSO is particularly attractive due to its high dielectric constant [17,18], strong solvating power for metal salts [19], and relatively low toxicity compared with other organic solvents [20]. We, therefore, hypothesise that adding a small fraction of DMSO (<2 wt.%) to a standard alkaline nickel ELD bath will swell surface-proximal nylon-6,6 chains, increasing PDA/TA adhesion and catalytic site density; promote nanocrystalline, low-stress nickel shells that better accommodate fibre deformation; and translate these interfacial refinements into superior thermal, mechanical, and electrochemical durability of the polymer–metal composite. Given the inherent versatility of electroless deposition techniques for conformal metal coating on complex geometries, the DMSO-modified ELD strategy demonstrated in this study offers promising avenues for broader application. By adjusting bath composition and surface functionalisation, this approach may be extended to other metals (e.g., Cu, Ag, and Co-P) and polar polymer substrates such as polyamide-11/12 or thermoplastic polyurethane (TPU). The ability to create durable, nanostructured metal layers on flexible polymer scaffolds also opens possibilities in the field of energy storage. Recent work by Wu et al. [21] reported electroless copper plating on polyimide scaffolds to fabricate 3D current collectors for lithium–metal batteries, achieving significant improvements in energy density and electrochemical stability. These findings underscore the broader potential of solvent-engineered metallisation strategies, such as the one proposed here, for creating robust, electrochemically active polymer–metal interfaces in wearable electronics, soft sensors, and energy storage systems. While DMSO was chosen in this study for its strong solvating power, moderate toxicity, and lower surface tension, further exploration of alternative polar aprotic solvents could provide additional tunability of coating properties, provided their compatibility with textile processing and environmental constraints. Figure 1 illustrates a potential application scenario, demonstrating how these DMSO-modified nickel-coated yarns could be effectively integrated into advanced electronic textile systems. Through direct comparison with conventional aqueous-based deposition, this research provides both fundamental insights into the role of solvent environments in electroless plating processes and practical strategies for optimising metal-coated polymeric substrates. The outcomes of this study hold significant implications for future developments in functional textiles, wearable electronics, and related high-performance coating technologies.

## 2. Materials and Methods

### 2.1. Materials

The nylon-6,6 yarns used in this study were 2-ply nylon-6,6 yarns, with each ply having a denier of 235 (Somac Threads Ltd., Chester, UK). Warp-knitted structure was chosen for some of the morphological analysis and functional evaluation. Specifically, the fabric contains 80% nylon-6,6 and 20% elastane (Plush Addict, Enderby, UK). Pure nylon 6,6 yarns were used for all morphological, compositional, crystallographic, and thermal characterisations in this study. The 80% nylon/20% spandex blend was used only in the electromechanical strain-sensing tests, as it provided the stretchability required for evaluating sensing performance under large deformations.

The chemicals used for pretreatment included dopamine hydrochloride ((HO)_2_C_6_H_3_CH_2_CH_2_NH_2_)·HCl, ≥98%, Sigma-Aldrich, Dorset, UK, Tris base (NH_2_C(CH_2_OH)_3_), Merck, Dorset, UK, tannic acid (C_76_H_52_O_46_), Merck, and ammonium tetrachloropalladate (II) ((NH_4_))_2_PdCl_4_, 97%, Sigma-Aldrich. All chemicals were of analytical grade.

The chemicals used for the nickel deposition process included nickel (II) sulphate hexahydrate ((NiSO_4_·6H_2_ONiSO), ≥98%, Merck), tri-sodium citrate dihydrate ((HOC(COONa)(CH_2_COONa)_2_·2H_2_O), Merck), lactic acid ((CH_3_CH(OH)COOH), Merck), and ammonia solution (35%, (NH_3_·H_2_O), Fisher Scientific, Loughborough, UK) for pH adjustment. The polar aprotic solvent dimethyl sulfoxide (DMSO, CH_3_SOCH_3_, Merck) was used to modify the deposition bath. Borane dimethylamine complex (DMAB, (CH_3_)_2_NH·BH_3_, 97%, Merck) was employed as the reducing agent.

All chemicals were used as received without further purification, and DI water was used as the solvent in all experiments.

### 2.2. Pretreatment of Nylon-6,6 Yarns

Before pretreatment, the nylon-6,6 yarns were washed in ethanol using an ultrasonic cleaner for 1 h to remove any surface contaminants. After ultrasonic cleaning, the yarns were thoroughly rinsed with deionised (DI) water and dried.

Given the hydrophobic nature of nylon-6,6, surface modification is a crucial step to enhance wettability and ensure a uniform coating. The first step involved immersing the yarns in a PDA solution prepared in Tris buffer. This solution was prepared by dissolving 1.2 g of Tris base in 1 L of DI water, and the PDA solution was prepared at a concentration of 20 mg/mL of dopamine hydrochloride in the Tris buffer. It facilitated the formation of a thin, adherent PDA layer on the nylon-6,6 surface, a critical aspect of the process. This PDA coating is formed through the self-polymerisation of dopamine in an alkaline environment provided by the Tris buffer, which maintains a pH of around 8.5. The yarns were soaked in this solution for 24 h and then rinsed with DI water and dried.

Subsequently, the yarns were immersed in a tannic acid solution (20 mg/mL in DI water) for 1 h. Tannic acid, a naturally occurring polyphenol, adsorbed onto the PDA-coated nylon-6,6 yarns through hydrogen bonding and hydrophobic interactions, further enhancing the surface’s ability to bind metal ions. Following the tannic acid treatment, the yarns were rinsed again with DI water and dried. The combined effect of PDA + Tris and tannic acid treatments provides a robust surface modification strategy for nylon-6,6 yarns.

### 2.3. Immobilisation of Catalysts

The next step involved immersion in an ammonium tetrachloropalladate (II) (NH_4_)_2_PdCl_4_) solution (1.422 mg/mL DI water) for 1 h to act as a catalyst. Ammonium tetrachloropalladate (II) served as a catalyst by adsorbing palladium ions onto the surface of the yarns. These Pd^2+^ ions were subsequently reduced to metallic palladium (Pd^0^), creating active catalytic sites that facilitate the reduction of nickel ions to metallic nickel during deposition. This catalytic activity ensured a successful nickel coating. After this catalytic treatment, the yarns were rinsed with DI water and dried, ready for nickel coating.

### 2.4. Metallisation by DMSO-Modified ELD

The metallisation process for the pretreated nylon-6,6 yarns was conducted using an aqueous-based ELD bath modified with varying concentrations of DMSO, a polar aprotic solvent. The ELD bath was prepared with a composition of 1 L of DI water as the solvent, 40 g of nickel (II) sulphate hexahydrate as the nickel ion source, 20 g of tri-sodium citrate dihydrate as a chelating agent to stabilise nickel ions in solution, and 20 g of lactic acid to enhance bath stability and adjust the chemical environment for deposition. The pH of the solution was adjusted to 10 using 35% ammonia solution to create optimal conditions for nickel ion reduction and deposition.

To explore the influence of DMSO on the nickel deposition process and coating properties, DMSO was added to the ELD bath in varying concentrations of 0.5 wt.% and 1 wt.% relative to the total nickel solution. These corresponded to adding 0.5 mL and 1 mL of DMSO per 100 mL of the bath solution. The introduction of DMSO aimed to investigate its role in altering the deposition kinetics, particularly its effect on nucleation and coating growth dynamics, given its known ability to modify solvation environments and surface energies. After adding DMSO, the solution was thoroughly mixed to ensure homogeneity before proceeding with nickel deposition.

The reducing agent, DMAB, was added to the bath at 1 g per 100 mL concentration as the final step to activate the reduction of nickel ions (Ni^2+^) to metallic nickel (Ni^0^). The pretreated and catalysed nylon-6,6 yarns were then immersed in the deposition bath. The coating process was carried out at room temperature (21–25 °C). Upon the completion of the coating process, the nickel-coated yarns were removed from the bath, thoroughly rinsed with DI water to eliminate residual chemicals, and dried in a vacuum oven at 60 °C for 12 h. The resultant nickel coatings were examined to evaluate their thickness, uniformity, and mechanical properties, particularly how DMSO influenced the coating’s structure. This process aimed to optimise the balance between coating flexibility and mechanical stability, providing insights into the role of DMSO in enhancing the overall performance of nickel-coated nylon-6,6 yarns. The schematic process of the experiment is shown in Figure S1.

### 2.5. Characterisation

Scanning electron microscopy (SEM) images and energy-dispersion X-ray (EDX) patterns were acquired using Zeiss Merlin FEG-SEM (Carl Zeiss Microscopy, Jena, Germany), equipped with an X-Max 150 EDX detector. X-ray diffraction (XRD) patterns were acquired using Bruker D8 Discover Autochanger (Bruker AXS GmbH, Karlsruhe, Germany). Transmission electron microscopy (TEM), scanning transmission electron microscopy (STEM), and STEM-EDX mapping were performed using Talos F200X (Thermo Fisher Scientific, Hillsboro, OR, USA) equipped with a Super-X EDX detector. Focused ion beam (FIB) cross-sectional samples were prepared using FEI Helios 660 (Thermo Fisher Scientific, Hillsboro, OR, USA) combined with the ex situ lift-out method (EXpressLO, Tescan Group, Lehigh Acres, FL, USA) to ensure the accuracy of the elemental mapping.

### 2.6. Electromechanical and Thermal Tests

Mechanical tests and electrical calibration of the strain sensor were performed using a universal testing machine (Instron 3344, Instron, Buckinghamshire, UK) and a multimeter (Keithley 2000, Keithley Instruments, Solon, OH, USA), respectively. The sensor dimensions were 2 cm × 1 cm, with copper wire electrodes used for connection to the multimeter.

Thermogravimetric analysis (TGA) was carried out to evaluate the thermal stability and decomposition behaviour of the coated fibres. The analysis was performed on a TA Instruments TGA Q500 (Leatherhead, UK) under an argon atmosphere. Samples were heated from ambient temperature (~20 °C) to 800 °C at a heating rate of 10 °C/min, and weight loss was recorded as a function of temperature.

### 2.7. Contact Angle Test

The surface wettability of the samples was evaluated using static water contact angle measurements performed on a Krüss Drop Shape Analyser (DSA100, KRÜSS GmbH, Hamburg, Germany). Deionised water droplets (3 µL) were carefully dispensed onto the fabric surface using a precision dosing system. Images were captured immediately upon droplet contact with the sample to minimise evaporation and dynamic spreading effects. The contact angle was measured using the instrument’s built-in image analysis software by fitting the droplet profile using a sessile drop method. Three representative fabric types were tested: pristine Lycra, aqueous-based electroless nickel-coated Lycra (A-ELD), and 1 wt.% DMSO-modified electroless nickel-coated Lycra.

### 2.8. Corrosion Test

The corrosion test was conducted using a standard three-electrode system. The working electrode consisted of a Ni-coated yarn (1 cm in length). An Ag/AgCl electrode served as the reference electrode, and a platinum electrode was used as the counter electrode. A 3.5 wt.% NaCl solution was employed as the electrolyte to simulate a corrosive environment. Electrochemical impedance spectroscopy, open circuit potential, and polarisation curves were plotted using an Autolab Metrohm 302 N (Metrohm Autolab, Utrecht, The Netherlands) electrochemical workstation. Polarisation curves were recorded both prior to corrosion testing and after immersing the samples in the electrolyte for 30 min. The polarisation scans were performed at a rate of 0.01 V/s with a step size of 0.001 V.

### 2.9. DMA Creep Behaviour Test

For the creep behaviour of the ELD Ni-coated yarns (1 cm in length), they were evaluated under −20 °C and 50 °C environments. Under each temperature, the samples were isothermal for 5 min and then displaced with 25 MPa for 200 min and recovered for 400 min.

## 3. Results

### 3.1. Morphological and Elemental Analysis

Given the hydrophobic nature of nylon-6,6, surface activation is essential for electroless deposition on polymeric substrates to enable metal nucleation and ensure coating continuity. In this study, a dual-step bio-inspired polymerisation strategy was employed using PDA and TA to create a chelation-rich interface. Upon immersion in mildly alkaline Tris buffer, dopamine undergoes oxidative self-polymerisation, forming a conformal PDA layer with catechol and amine functionalities that are capable of binding metal ions and reducing agents. The PDA layer offers functional groups for further modifications, while the TA layer enhances the binding and stabilisation of metal ions or nanoparticles. This combination is essential for the subsequent deposition of nickel in the electroless nickel plating process. The importance of dual anchoring is further demonstrated in Figure S2, indicating that PDA-only and TA-only treatments result in cracked, non-uniform nickel coatings, while the PDA/TA-treated fibres exhibit a smooth, continuous surface.

**Figure 2:**
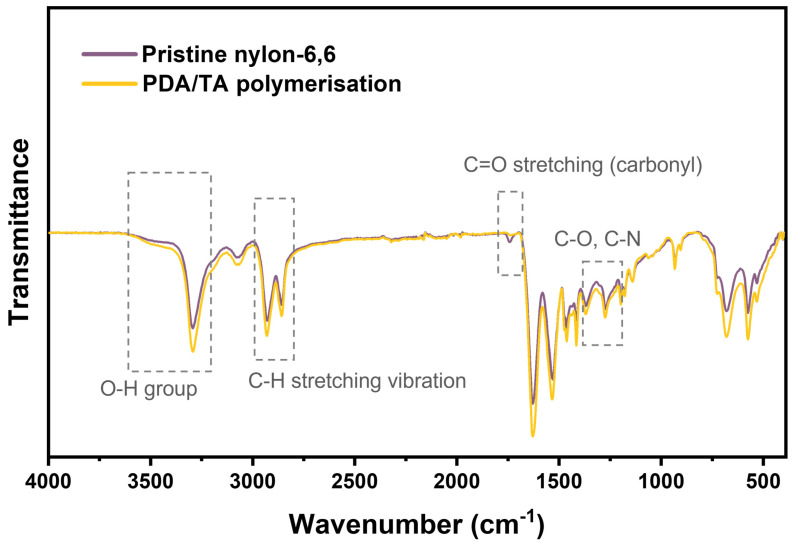
FT-IR spectra of pristine nylon-6,6 and after polymerisation treatment.

FT-IR in Figure 2 confirms that the catechol–phenolic coating profoundly alters the surface chemistry of the fibre. Relative to pristine nylon-6,6, the treated sample displays a broad, intensified envelope at 3200–3500 cm^−1^ that arises from overlapping O-H and N-H stretches, signalling a high density of phenolic and amine groups supplied by polydopamine and tannic acid. New aromatic C-H and C=C vibrations emerge at 2850–3000 cm^−1^ and ~1510 cm^−1^, respectively, while a markedly stronger carbonyl band at 1700–1730 cm^−1^ reflects esterified galloyl units and quinone forms generated during oxidative polymerisation. Collectively, these spectral changes verify the formation of a catechol-rich layer that replaces the low-polarity amide surface with a highly polar interface capable of hydrogen bonding and metal–ion chelation.

**Figure 3:**
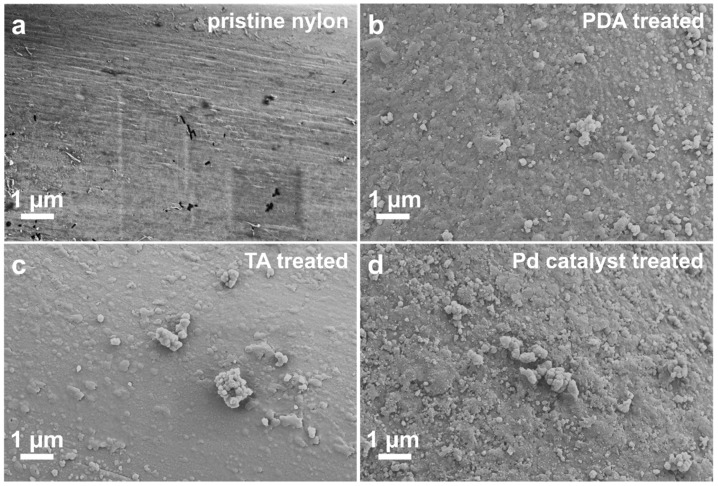
SEM images of nylon-6,6 yarn surfaces in different stages of surface modification: (**a**) pristine nylon-6,6 yarn; (**b**) yarn treated with PDA for 24 h; (**c**) PDA-coated yarns subjected to 1 h TA treatment in addition to the 24 h treatment; (**d**) catalyst-immobilised yarns after polymerisation treatment.

The corresponding SEM images in Figure 3 illustrate the progressive evolution of the nylon-6,6 surface morphology following each stage of the surface functionalisation process. The untreated fibre (Figure 3a) displays a smooth, longitudinally grooved surface characteristic of melt-spun nylon-6,6, offering limited sites for heterogeneous nucleation. Following PDA treatment (Figure 3b), the surface becomes uniformly coated with hemispherical nodules, indicative of self-polymerised PDA aggregates that impart nanoscale roughness. Subsequent deposition of TA results in the formation of a finer particulate matrix interspersed with occasional larger clusters (Figure 3c), further enhancing the surface area and catechol group density. Finally, immersion in an ammonium tetrachloropalladate (II) solution leads to the immobilisation of a dense and well-dispersed population of palladium nanoparticles across the textured surface (Figure 3d), establishing a high density of catalytic nucleation sites essential for subsequent electroless nickel deposition.

**Figure 4:**
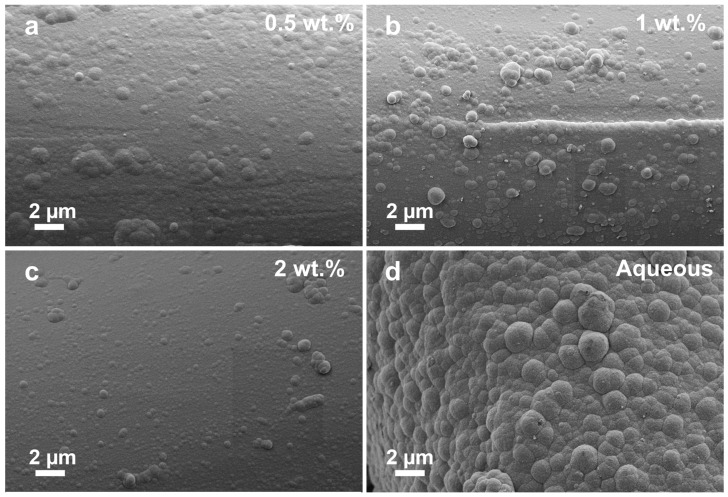
Comparative SEM images of (**a**) 0.5 wt.% DMSO ELD, (**b**) 1 wt.% DMSO ELD, (**c**) 2 wt.% DMSO ELD, and (**d**) 100% aqueous-based ELD.

Figure 4 presents comparative SEM images showing the surface morphology of nickel-coated nylon-6,6 yarns obtained from ELD baths with different DMSO concentrations in comparison to the conventional fully aqueous-based ELD (A-ELD) bath. At a lower DMSO concentration (0.5 wt.%, Figure 4a), the coating surface shows moderate smoothness with evident nodular structures and some larger agglomerates. Upon increasing the DMSO content to 1 wt.% (Figure 4b), a clear improvement in coating morphology is observed; the nickel deposits become notably smoother, exhibiting fewer large agglomerates, a finer grain structure, and enhanced surface homogeneity. This refinement is even more pronounced at 2 wt.% DMSO concentration (Figure 4c), resulting in the smoothest and most uniform surface of the DMSO-modified coatings, featuring fewer defects. However, beyond 2 wt.% DMSO, only marginal additional improvements are noted, indicating a possible upper threshold for solvent benefits. Further investigation into higher DMSO concentrations (3–9 wt.%) reveals a decline in coating quality, with increased surface roughness, localised agglomeration, and poor adhesion at ≥7 wt.%, as shown in Figure S3, highlighting the importance of optimising solvent concentration in ELD. In contrast, the fully aqueous-based ELD bath (Figure 4d) exhibits a noticeably rougher and coarser surface morphology. Large spherical nickel nodules dominate the surface, creating an uneven coating with significant microstructural irregularities and apparent internal stress.

**Figure 5:**
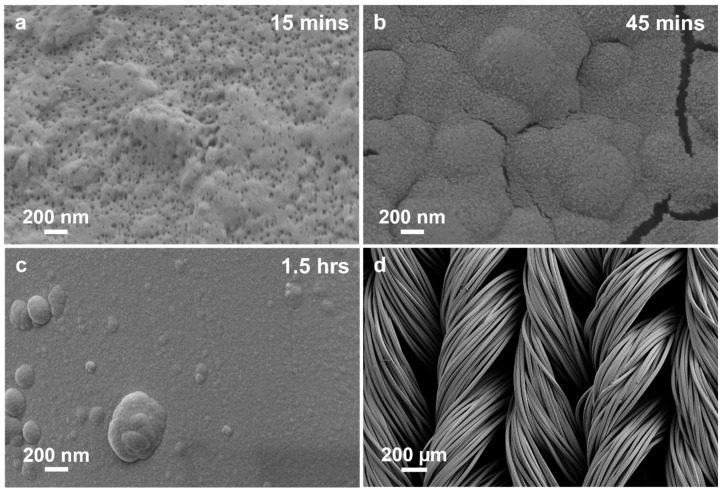
(**a**–**c**) SEM images of the evolution of the 1 wt.% DMSO ELD Ni-coating at different times and (**d**) Ni-coated knitted fabric after 1.5 h ELD.

Figure 5 provides insight into the progressive morphological evolution of nickel coatings deposited from the 1 wt.% DMSO-modified electroless nickel bath over different deposition durations. At the early deposition stage of 15 min (Figure 5a), the nylon-6,6 surface exhibits a dense distribution of fine nickel nuclei, indicative of rapid nucleation. This initial coating layer appears porous with numerous small gaps, highlighting the discrete nature of early-stage nickel nucleation and growth influenced by the DMSO-modified deposition kinetics. As the deposition proceeds to 45 min (Figure 5b), noticeable grain coalescence occurs, forming larger, rounded nickel nodules that create a continuous yet still irregular coating. At this intermediate stage, some surface cracking is observed, likely arising from internal stresses developed during the progressive coalescence and densification of the nickel grains. By 1.5 h of deposition (Figure 5c), the coating morphology suggests further refinement, demonstrating a smoother and more uniform surface with significantly fewer defects. The reduced presence of larger nodules and surface irregularities indicates effective stress relaxation and grain refinement achieved by prolonged interaction with the DMSO-containing bath.

Figure 5d provides a lower-magnification SEM image of the knitted fabric after 1.5 h of deposition, revealing uniform nickel coverage throughout the textile structure. This confirms that the DMSO-modified bath enables efficient and homogeneous coating even on complex textile architectures, achieving good coverage without significantly compromising the flexibility and integrity of the underlying nylon-6,6 fibres. Figure S4 SEM images at progressively lower magnification confirm the uniformity and structural integrity of the 1 wt.% DMSO-modified electroless nickel coating. At high magnification (Figure S4a), the coating exhibits dense nanograins with full surface coverage and no visible defects. Intermediate views (Figure S4a–c) show conformal coating across individual filaments and filament bundles, with consistent texture even in crossover regions. The lowest magnification (Figure S4d) confirms the preservation of the yarn’s macrostructure without fibre fusion or deformation, demonstrating the coating’s compatibility with flexible textile architectures.

**Figure 6:**
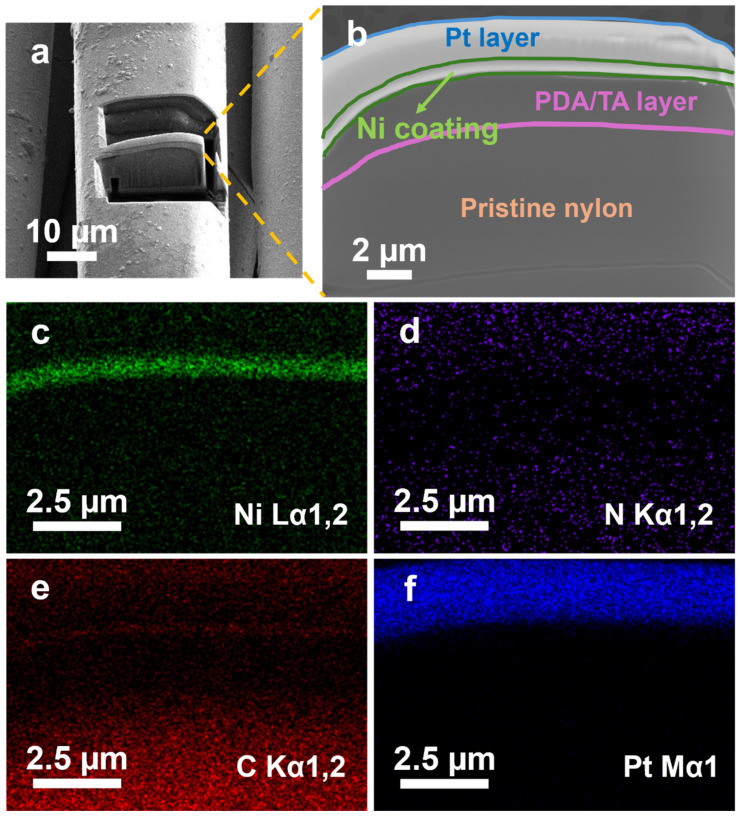
Composition of 1 wt.% DMSO ELD nickel-coated yarn: (**a**) SEM image of preparation of FIB sample; (**b**) FIB cross-sectional SEM image of (**a**); (**c**–**f**) associated elemental mappings of (**c**) nickel (Ni Lα), (**d**) nitrogen (N Kα), (**e**) carbon (C Kα), and (**f**) platinum (Pt Mα).

Figure 6 provides a cross-sectional view of the 1 wt.% DMSO-modified Ni coating on nylon-6,6 fibres via focused ion beam (FIB) milling and subsequent SEM/EDX mapping. Compared to the top-surface EDX maps presented in Figure S5, which confirm the distribution of nickel (Ni Lα), carbon (C Kα), nitrogen (N Kα), and oxygen (O Kα) at the fibre surface, the cross-sectional image in Figure 6 offers additional insight into coating thickness and multi-layer interfaces. Figure 6a shows the SEM overview image of the yarn after the FIB milling process, clearly indicating the precise location of the cross-sectional analysis. Figure 6b highlights a cross-sectional cut, revealing the original nylon-6,6 substrate, an intermediate PDA/TA layer, the overlying Ni coating, and a protective platinum (Pt) layer deposited during the FIB sample preparation.

The corresponding elemental maps (Figure 6c–f) verify the strong Ni signal at the outer layer, while carbon- and nitrogen-rich regions remain concentrated in the underlying polymer and functional layer. The strong, sharply defined nickel signal (Ni Lα, Figure 6c) closely matches the SEM-identified metallic layer, demonstrating homogeneous nickel distribution and coating uniformity. Nitrogen (N Kα, Figure 6d) and carbon (C Kα, Figure 6e) signals distinctly map to the PDA/TA functional layer and underlying nylon-6,6 substrate, clearly reflecting their polymeric organic nature. These organic elemental signals show minimal penetration into the metallic region, confirming a stable and chemically defined interface with no significant polymer degradation or metal contamination. The distinct Pt Mα signal in Figure 6f originates solely from the protective platinum layer deposited during sample preparation and delineates the sample surface. This cross-sectional elemental mapping not only confirms the presence and uniformity of the nickel coating but also verifies the structural integrity of the intermediate polymerisation layer that significantly contributes to enhanced adhesion between the metallic coating and the polymer substrate.

### 3.2. Crystallographic Structure Analysis

**Figure 7:**
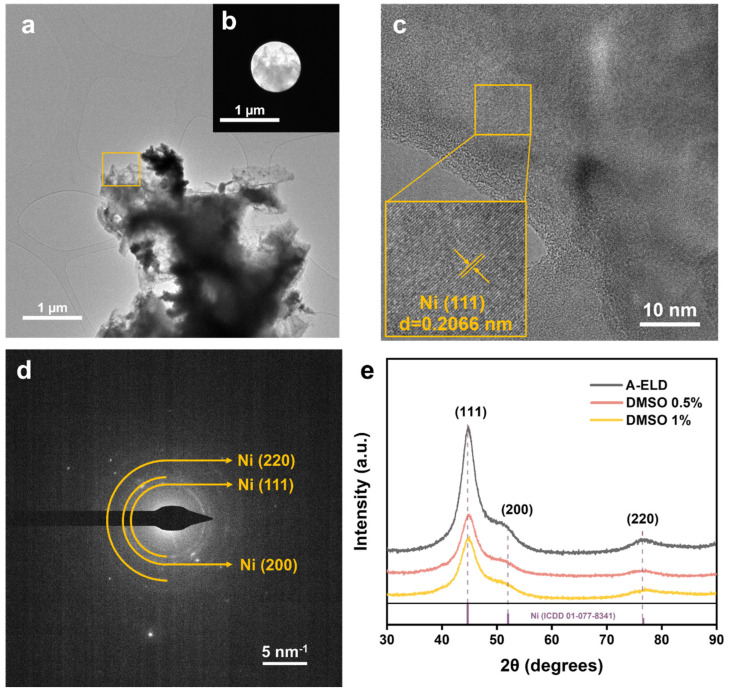
Microstructure of 1 wt.% DMSO ELD nickel coating: (**a**) TEM image; (**b**) bright-field image of the selected region from (**a**); (**c**) HRTEM image of (**a**); (**d**) SAED image; (**e**) XRD patterns of A-ELD, DMSO ELD, and reference peaks.

The crystallographic features of the electroless nickel coatings were evaluated through a combination of TEM, SAED, and XRD (Figure 7). Low-magnification TEM images (Figure 7a) reveal a predominantly nanocrystalline structure, while high-resolution TEM (HRTEM) micrographs (Figure 7b) confirm well-defined lattice fringes consistent with face-centred cubic (FCC) nickel. Measurements of the interplanar spacing in the HRTEM images (0.2066 nm) match the characteristic (111) planes for FCC Ni, verifying the formation of nickel crystallites.

In support of the TEM observations, the SAED pattern (Figure 7c) exhibits distinct diffraction rings corresponding to the (111), (200), and (220) planes of nickel, indicating that the deposited coatings are polycrystalline. Notably, the rings are relatively broad in DMSO-modified coatings, suggesting a fine-grained microstructure and a higher defect density. This stands in contrast to the sharper rings observed in the A-ELD sample, which point to larger grain sizes and a more highly ordered lattice. XRD analysis (Figure 7d) further underscores the effect of DMSO on nickel crystallinity. While the A-ELD sample exhibits prominent, narrow peaks—particularly at (111)—the DMSO-containing samples display peak broadening and reduced intensity. This broadening becomes more pronounced with increasing DMSO concentration, indicative of smaller crystallite size and a decrease in long-range order. Such nanocrystalline structures typically arise when the nucleation rate is elevated relative to grain growth, a scenario plausibly driven by DMSO’s ability to alter ion mobility, reduce supersaturation, and slow down metal agglomeration in solution.

Crucially, the reduced crystallite size in DMSO-modified coatings can have both advantages and trade-offs. On the one hand, finer grains often translate to improved mechanical flexibility, enhanced strain sensitivity, and greater thermal stability due to the grain-boundary-mediated diffusion pathways. On the other hand, the loss of strong (111) preferential orientation and the presence of more grain boundaries may marginally affect other properties, such as electrical conductivity or hardness. Nonetheless, the nanocrystalline morphology achieved through DMSO incorporation appears well-suited for applications in wearable electronics and advanced textiles, where flexibility and durability are paramount. By revealing how DMSO drives a shift from a highly crystalline, coarse-grained nickel to a nanocrystalline structure, this analysis highlights the critical role of solvent engineering in controlling crystallographic features during electroless deposition. Coupled with morphological and compositional evidence, these findings offer new insights into optimising ELD baths to tailor the mechanical, thermal, and electrical performance of nickel-coated polymers.

## 4. Performance Evaluation

**Figure 8:**
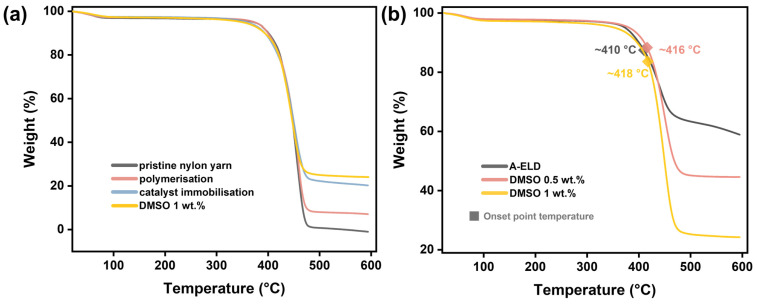
(**a**) TGA curves of nylon-6,6 yarns at different stages of treatment and nickel coating; (**b**) TGA curves of A-ELD and DMSO-modified ELD Ni-coated yarns.

Figure 8 presents the thermogravimetric analysis (TGA) curves acquired under an argon atmosphere. Figure 8a demonstrates pristine nylon-6,6 yarns and those subjected to sequential treatment steps, including PDA/TA polymerisation, catalyst immobilisation, and final electroless nickel deposition using a 1 wt.% DMSO-modified bath. The analysis provides a clear indication of the incremental mass addition onto the nylon-6,6 substrate at each processing stage. The pristine nylon-6,6 yarn exhibits near-total thermal degradation by 500 °C, with negligible residual weight, reflecting the complete decomposition of the organic matrix. Following polymerisation, a measurable increase in residual mass to 6.89% is observed, confirming successful surface modification with organic functional layers. Catalyst immobilisation further increases the residue to 17.59%, attributable to the anchoring of metallic precursors onto the polymerised surface. After nickel coating via the DMSO-modified ELD bath, the residual weight rises to 24.09%, indicating substantial incorporation of metallic nickel onto the yarn surface. Based on this mass increase and geometric estimates of the yarn bundle, the average nickel coating thickness was calculated to be approximately 65 nm, which aligns with morphological observations in Figure 4.

Figure 8b presents a focused comparison of thermal properties for yarns coated with A-ELD versus DMSO-modified baths at 0.5 wt.% and 1 wt.% concentrations. The A-ELD sample shows the highest residual weight, indicative of a thicker nickel coating, but it possesses the lowest onset decomposition temperature (approximately 411 °C). This reduced thermal stability can be attributed to its coarser, polycrystalline morphology, characterised by higher defect densities and internal stresses, facilitating easier thermal degradation pathways. Conversely, yarns coated from baths containing DMSO show improved onset decomposition temperatures, specifically at approximately 416 °C (0.5 wt.% DMSO) and 418 °C (1 wt.% DMSO). The enhancement in thermal stability in these samples can be directly linked to their refined nanocrystalline microstructure, which reduces internal stresses and defect densities and provides fewer pathways for thermal degradation and oxidation processes to propagate.

**Figure 9:**
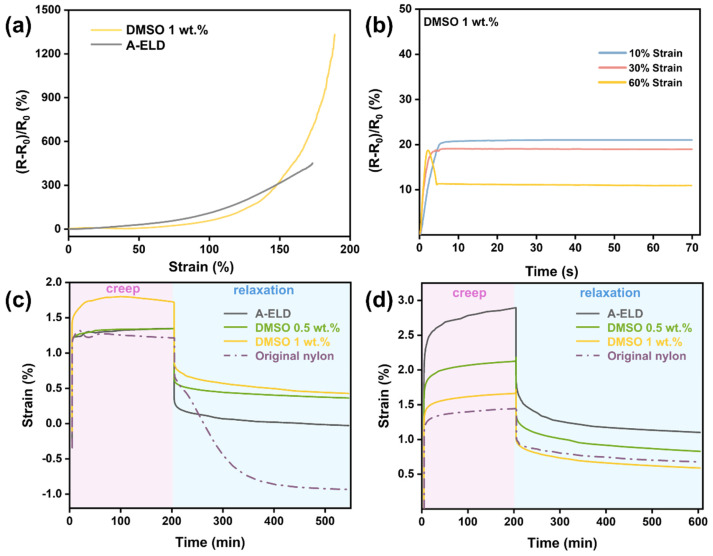
The 1 wt.% DMSO Ni-coated (**a**) Lycra knitted fabric electrical response to strain; (**b**) the relative resistance changes in the strain sensor with gradient strains to show the static stability of Lycra knitted fabric; (**c**) nylon-6,6 yarns’ creep testing curves at −20 °C environment; (**d**) nylon-6,6 yarns’ creep testing curves at 50 °C environment.

All strain-sensor measurements were conducted on warp-knitted fabrics made from the 80% nylon-6,6/20% elastane yarns that were metallised in the same 1 wt.% DMSO-modified electroless nickel bath. Figure 9a,b show the electromechanical response of nickel-coated Lycra knitted fabric, demonstrating its suitability for strain-sensing applications. In Figure 9a, the electrical response to tensile deformation exhibits a highly sensitive and non-linear increase in relative resistance, reaching over 1400% at 200% strain. Below an approximately 150% strain, the DMSO-modified coating exhibits a smaller resistance change because its compliant nanocrystalline network delays crack formation. Once this threshold is surpassed, a rapid increase in ΔR/R_0_ (>1200%) is observed, giving the film a broader working range and a higher peak gauge factor than the A-ELD control. This pronounced change reflects the exceptional strain sensitivity of the coating, which likely results from microstructural rearrangements and crack propagation in the nanocrystalline nickel layer under elongation. Such a sharp, tuneable resistance response is advantageous for high-resolution strain sensing in stretchable electronics. Figure 9b exhibits the electrical stability of the coated fabric under fixed strain levels of 10%, 30%, and 60%. At lower strain levels (10% and 30%), the relative resistance shows consistent and stable responses over time. Under 60% strain, the resistance initially decreases slightly before rising and stabilising. This behaviour is attributed to the nature of the knitted fabric structure: As the loops are stretched, they begin to straighten and align, improving contact between conductive regions and momentarily reducing resistance. Once the loops are fully extended and additional strain begins to pull on the individual yarns, the conductive pathways become increasingly disrupted, resulting in a rise in resistance. This two-stage electrical response, with an initial decrease followed by a sharp increase, is characteristic of conductive coatings applied to knitted elastic structures and reflects the interplay between mechanical deformation modes and current pathways in textile-based sensors. Nonetheless, the results confirm that the 1 wt.% DMSO Ni coating retains functional conductivity and responsiveness even under substantial mechanical deformation, validating its potential use in wearable sensing platforms.

Creep tests were performed at both 50 °C and −20 °C (Figure 9c,d) to gauge the coatings’ mechanical stability under different thermal conditions. At the elevated temperature (50 °C), the A-ELD sample shows the largest creep strain, reflecting poor mechanical stability likely caused by a coarse-grained, defect-rich microstructure that cannot resist sustained deformation at elevated temperature. In comparison, both DMSO-modified samples, especially the 1 wt.% DMSO coating, exhibit significantly lower creep strain and improved dimensional stability. The 1 wt.% DMSO sample also shows smoother recovery behaviour with minimal permanent deformation, highlighting its enhanced thermal robustness. Meanwhile, the original nylon-6,6 again shows minimal strain but lacks sufficient recovery, indicating the limits of the bare substrate under thermal load. At sub-zero temperatures (−20 °C), the trend partially reverses. Here, the DMSO-modified samples reveal relatively higher creep strain compared to A-ELD, suggesting that the finer-grained structure, while beneficial at higher temperatures, may also impart increased ductility at lower temperatures. This underscores a fundamental trade-off between flexibility and stability that arises from microstructural refinement. The choice of optimal DMSO concentration thus depends on the specific temperature and mechanical demands of the intended application.

**Figure 10:**
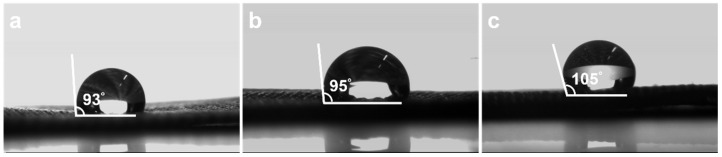
Contact angle images comparing the wettability of (**a**) pristine Lycra substrate, (**b**) aqueous-based electroless Ni-coated Lycra (A-ELD), and (**c**) 1 wt.% DMSO-modified electroless Ni-coated Lycra. The measured contact angles were 93°, 95°, and 105°, respectively.

The static-water contact angle images in Figure 10 quantify how electroless nickel deposition, and, in particular, solvent engineering with DMSO, modifies the surface wettability of the Lycra fabric. Nylon-6,6 can absorb up to 8.5 wt.% water at room temperature [22], reflecting its strong affinity for polar solvents due to amide group hydrogen bonding. In contrast, spandex typically absorbs <1.5 wt.% water [23]. This difference in water uptake supports the conclusion that swelling and solvent interaction during aqueous or DMSO-modified ELD is primarily driven by the nylon-6,6 component. Pristine Lycra exhibits a contact angle of 93° (Figure 10a), consistent with the moderate hydrophobicity expected for polyurethane-based fibres and the microscale roughness of the knit. Aqueous electroless deposition results in only a marginal change (95°, Figure 10b), indicating that the Ni film deposited from a fully aqueous bath inherits surface chemistry and topography that are similar in water affinity to the underlying textile. In contrast, the sample prepared using the 1 wt.% DMSO-modified bath shows a pronounced increase to 105° (Figure 10c), shifting the interface from moderately to distinctly hydrophobic (θ > 100°).

This increase of approximately 12° in contact angle is likely attributable to surface structural and chemical modifications induced by the presence of the DMSO co-solvent during electroless deposition. The incorporation of DMSO results in a more refined, uniform nanocrystalline nickel layer, which could decrease surface roughness and reduce capillary-driven wetting. Additionally, the presence of DMSO may alter the surface chemistry of the deposited nickel, potentially resulting in fewer hydroxylated surface sites. A reduction in hydroxyl groups would diminish hydrogen bonding interactions with water droplets, consequently elevating the apparent contact angle. Collectively, these structural and chemical surface modifications explain the observed enhancement in surface hydrophobicity.

**Figure 11:**
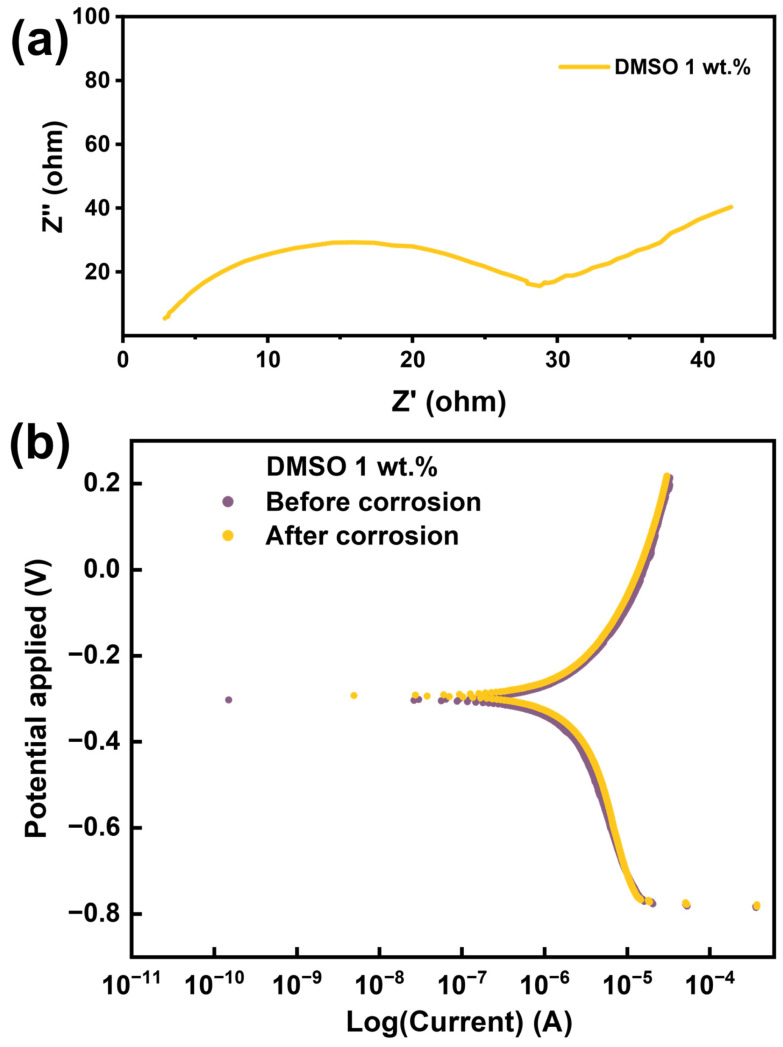
Electrochemical analysis of 1 wt.% DMSO-modified electroless nickel-coated nylon-6,6 yarns: (**a**) Nyquist plot from electrochemical impedance spectroscopy (EIS) showing charge transfer resistance characteristics of the coating; (**b**) potentiodynamic polarisation curves before and after accelerated corrosion testing.

Figure 11 evaluates the electrochemical stability of nickel coatings prepared using the 1 wt.% DMSO-modified ELD bath. The electrochemical impedance spectroscopy (EIS) response is shown in Figure 11a, and the potentiodynamic polarisation behaviour is presented in Figure 11b. For comparison, the corresponding polarisation response of the A-ELD sample is provided in Figure S6. In Figure 11a, the Nyquist plot of the 1 wt.% DMSO sample displays a broad semicircular arc, indicating a high charge transfer resistance (R_ct_). This response reflects the formation of a compact, low-defect nickel layer that effectively hinders electrochemical reactions at the coating–electrolyte interface. The absence of a low-frequency tail suggests good coating uniformity and minimal porosity, with limited diffusion of corrosive species through the film.

Figure 11b shows the polarisation curves of the DMSO-coated sample before and after an accelerated corrosion test. The minimal shift in both potential and corrosion current (I_corr_) after exposure indicates strong electrochemical durability. This stability points to the presence of a dense and adherent nanocrystalline nickel coating that resists corrosion initiation and propagation, even after extended environmental exposure. In contrast, the polarisation curves for the A-ELD sample in Figure S6 reveal a substantially greater deviation between the pre- and post-corrosion states. The corrosion current increases significantly, and the shift in corrosion potential is more pronounced, suggesting that the coating has undergone more severe degradation. This is consistent with the A-ELD sample’s previously observed coarse-grained structure, which provides more sites for corrosion to initiate, particularly at grain boundaries and interfacial defects. From a functional standpoint, the increased hydrophobicity of the DMSO-modified coating complements its superior electrochemical performance. A less wettable surface limits electrolyte penetration into grain boundaries and defect sites, helping to maintain the integrity of the passive oxide film and thereby enhancing corrosion resistance. At the same time, the fabric remains sufficiently hydrophilic (θ ≈ 105°) to allow occasional moisture exchange, which is advantageous for wearer comfort in e-textile applications.

## 5. Conclusions

This study demonstrates the significant potential of solvent engineering, specifically through incorporating DMSO into electroless nickel deposition baths for optimising polymer–metal interfacial properties on polymer substrates. Introducing DMSO into conventional aqueous nickel-plating baths significantly improved coating uniformity, surface morphology, and grain refinement, resulting in a robust, closely adherent nanocrystalline nickel layer. These enhancements directly arise from increased nylon-6,6 chain mobility and improved surface functionalisation induced by DMSO-assisted swelling.

The polymer–metal hybrid coatings obtained from the DMSO-modified baths exhibited substantial improvements in several key performance metrics essential for functional textile applications. Thermogravimetric analysis showed enhanced thermal stability, with elevated decomposition onset temperatures due to reduced internal stresses and defect minimisation. Mechanical tests, including creep measurements, demonstrated superior dimensional stability and mechanical resilience under elevated temperatures, underscoring the advantages of finer-grained nickel structures for maintaining integrity under thermal and mechanical stress. Surface wettability analysis indicated that the moderate hydrophobicity increase from DMSO incorporation notably correlates with improved electrochemical durability, as evidenced by higher charge transfer resistance and stable polarisation characteristics under corrosive conditions. The use of low-cost reagents and ambient, solution-based processing steps makes this method amenable to scale-up via existing textile-finishing infrastructure, supporting its potential for large-scale and cost-effective deployment.

## Data Availability

The original contributions presented in this study are included in the article/Supplementary Materials. Further inquiries can be directed to the corresponding authors.

## Supplementary Materials

The following supporting information can be downloaded at: https://www.mdpi.com/article/10.3390/polym17121693/s1, Figure S1. Schematic representation of the fabrication process for DMSO-modified ELD Ni-coated nylon yarns; Figure S2. SEM images of 1 wt.% DMSO-modified ELD nickel-coated nylon-6,6 under different polymerisation treatments. (a) PDA-only. (b) TA-only. (c) PDA/TA treated; Figure S3. SEM images of (a) 3 wt.%, (b) 4 wt.%, (c) 6 wt.%, (d) 7 wt.%, (e) 8 wt.%, (f) 9 wt.% DMSO-modified ELD Ni-coated nylon yarns; Figure S4. SEM images of 1 wt.% DMSO-modified electroless nickel-coated nylon yarn at increasing magnifications: (a) 70k ×, (b) 20k ×, (c) 6k ×, and (d) 2k ×; Figure S5. DMSO 1 wt.% modified ELD Ni-coated nylon yarns, (a) SEM image and (b–e) associated elemental mapping; Figure S6. The potentiodynamic polarisation curves of A-ELD Ni-coated nylon yarn.
