# Supplementary material for: Tuning Polymer–Metal Interfaces via Solvent-Engineered Electroless Nickel Coatings on Functional Fibres
Source: Polymers (Basel). 2025 Jun 18;17(12):1693. doi: 10.3390/polym17121693 (PMC12196621; doi:10.3390/polym17121693)
Supplement: Supplementary file 1 [file polymers-17-01693-s001.zip › polymers-3686625-supplementary.pdf]

Supporting Information

Tuning Polymer-Metal Interfaces via Solvent-Engineered Electroless Nickel Coatings on Functional Fibres

Chenyao Wang[1], Heng Zhai[2 *], Xuzhao Liu[1], David Lewis[2], Yuhao Huang[1], Ling Ai [1], Xinyi Guan[1, 3], Hugh Gong[1], Xuqing Liu[1 *], Anura Fernando[1 *]

[1]*Department of Materials, The University of Manchester, Manchester, M13 9PL, United Kingdom*

[2]*Department of Chemical Engineering, The University of Manchester, M13 9PL, United Kingdom*

[3]*Technical Textile Research Centre, University of Huddersfield, Huddersfield, HD1 3DH, United Kingdom*

[*]*Corresponding author: xqliu@nwpu.edu.cn (Xuqing Liu); heng.zhai@manchester.ac.uk (Heng Zhai); anura.fernando@manchester.ac.uk (Anura Fernando)*

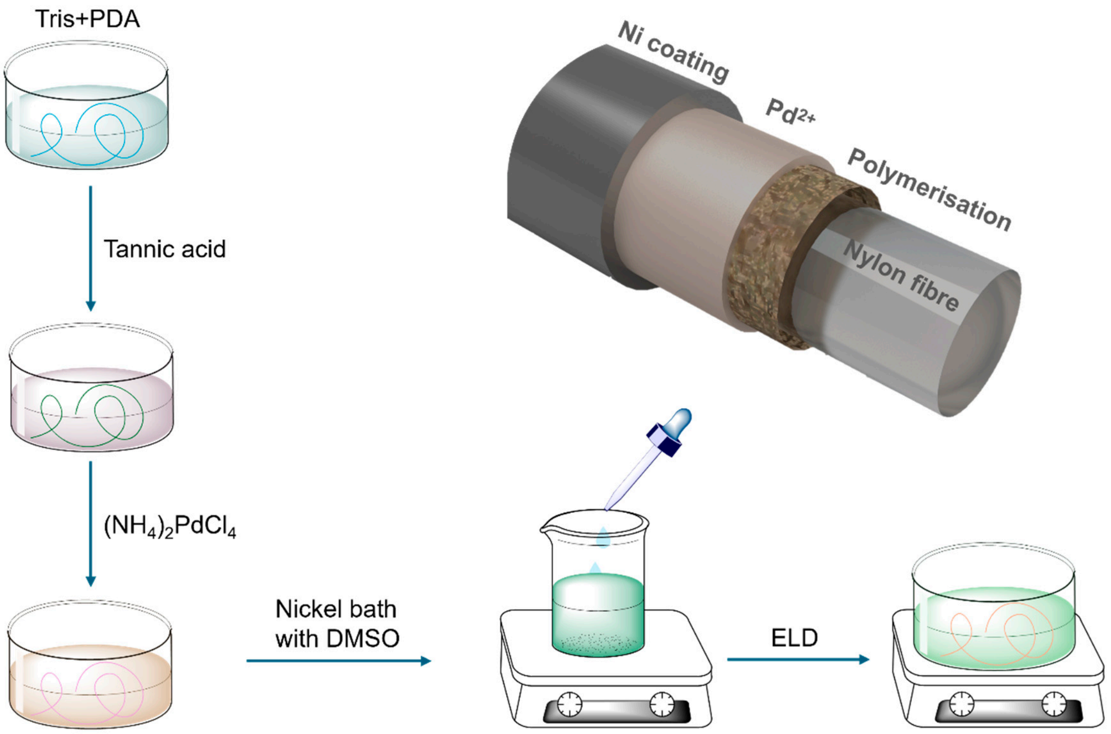

Figure S1. Schematic representation of the fabrication process for DMSO-modified ELD Ni-coated nylon yarns.

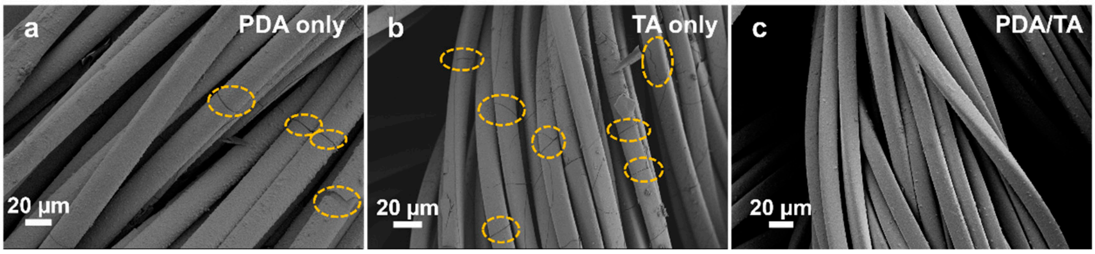

Figure S2. SEM images of 1 wt.% DMSO-modified ELD nickel-coated nylon-6,6 under different polymerisation treatments. (a) PDA-only. (b) TA-only. (c) PDA/TA treated.

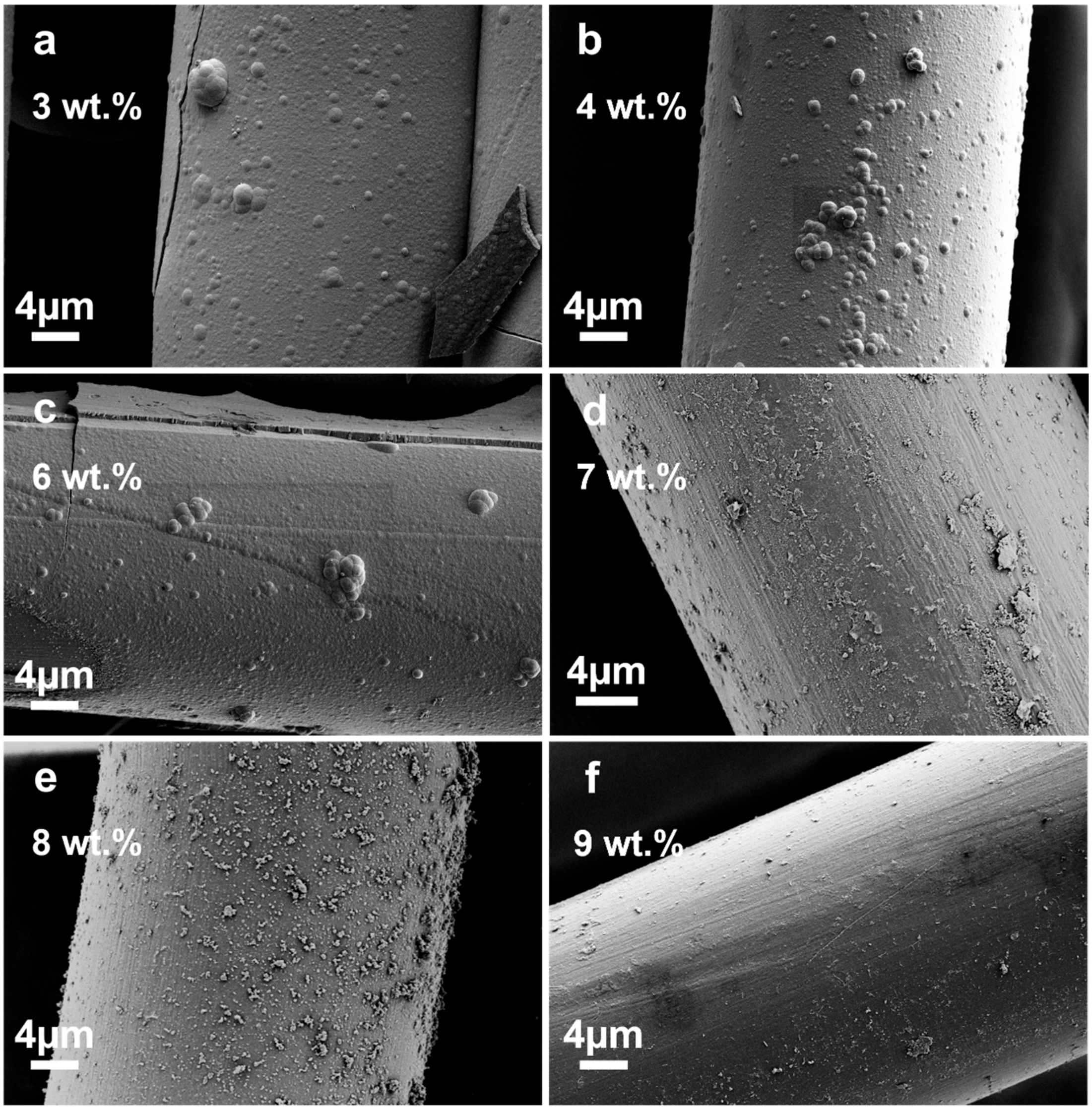

Figure S3. SEM images of (a) 3 wt.%, (b) 4 wt.%, (c) 6 wt.%, (d) 7 wt.%, (e) 8 wt.%, (f) 9 wt.% DMSO-modified ELD Ni-coated nylon yarns.

Figure S3 presents SEM images of nickel-coated nylon yarns deposited from electroless baths containing higher DMSO concentrations (3 - 9 wt.%). These images provide insight into the effect of increasing solvent content beyond the optimised range (0.5 - 2 wt.%) discussed in the main manuscript (**Figure 4**). At moderate DMSO levels (3 - 6 wt.%, **Figures S3a-S3c**), the coatings exhibit a mixture of localised agglomerates and partially coated regions. While some areas display continuous coverage, nodular growths and uneven deposition patterns are evident, suggesting that higher DMSO content begins to interfere with nickel nucleation and grain coalescence. Compared to the well-defined coatings seen at 1 - 2 wt.% DMSO

(**Figure 4**), these samples show greater surface roughness and inconsistencies in deposit thickness. At higher DMSO concentrations (⩾7 wt.%, **Figures S3d-S3f**), coating quality deteriorates significantly. The nickel deposits become increasingly dispersed, fragmented, and weakly adhered to the nylon fibres. Particularly at 9 wt.% DMSO (**Figure S3f**), the coating appears severely disrupted, with minimal metal coverage and visible detachment from the substrate. This suggests that excessive DMSO hinders nickel ion transport and adsorption, preventing stable film formation.

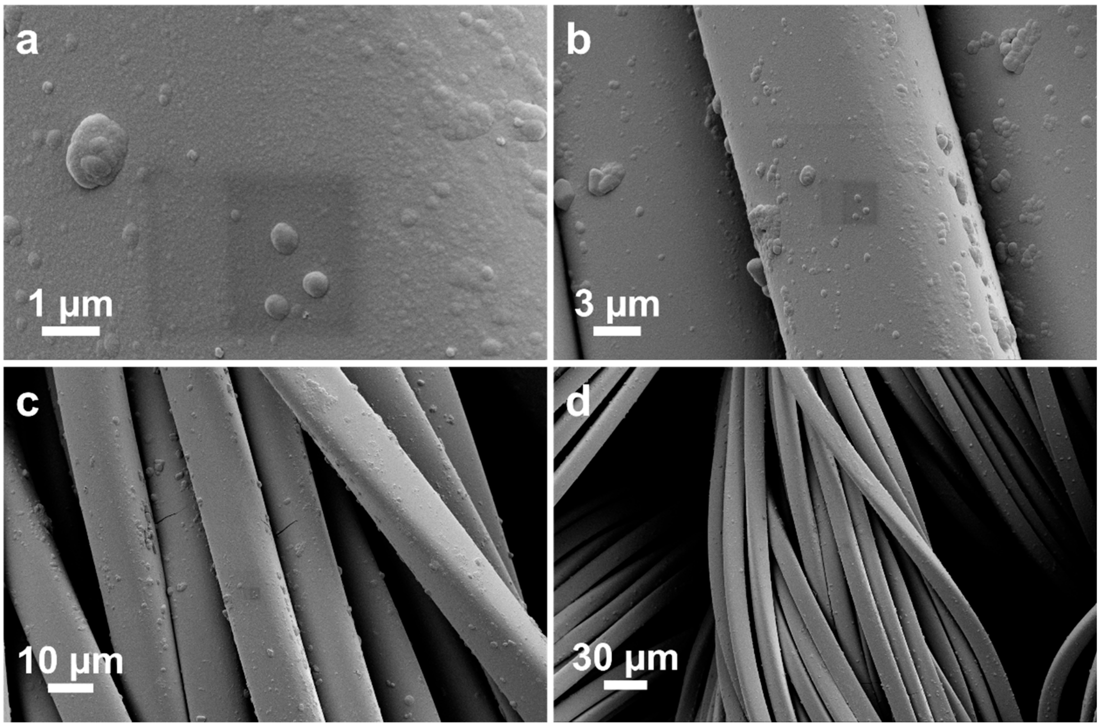

Figure S4. SEM images of 1 wt.% DMSO-modified electroless nickel-coated nylon yarn at increasing magnifications: (a) 70k ×, (b) 20k ×, (c) 6k ×, and (d) 2k ×.

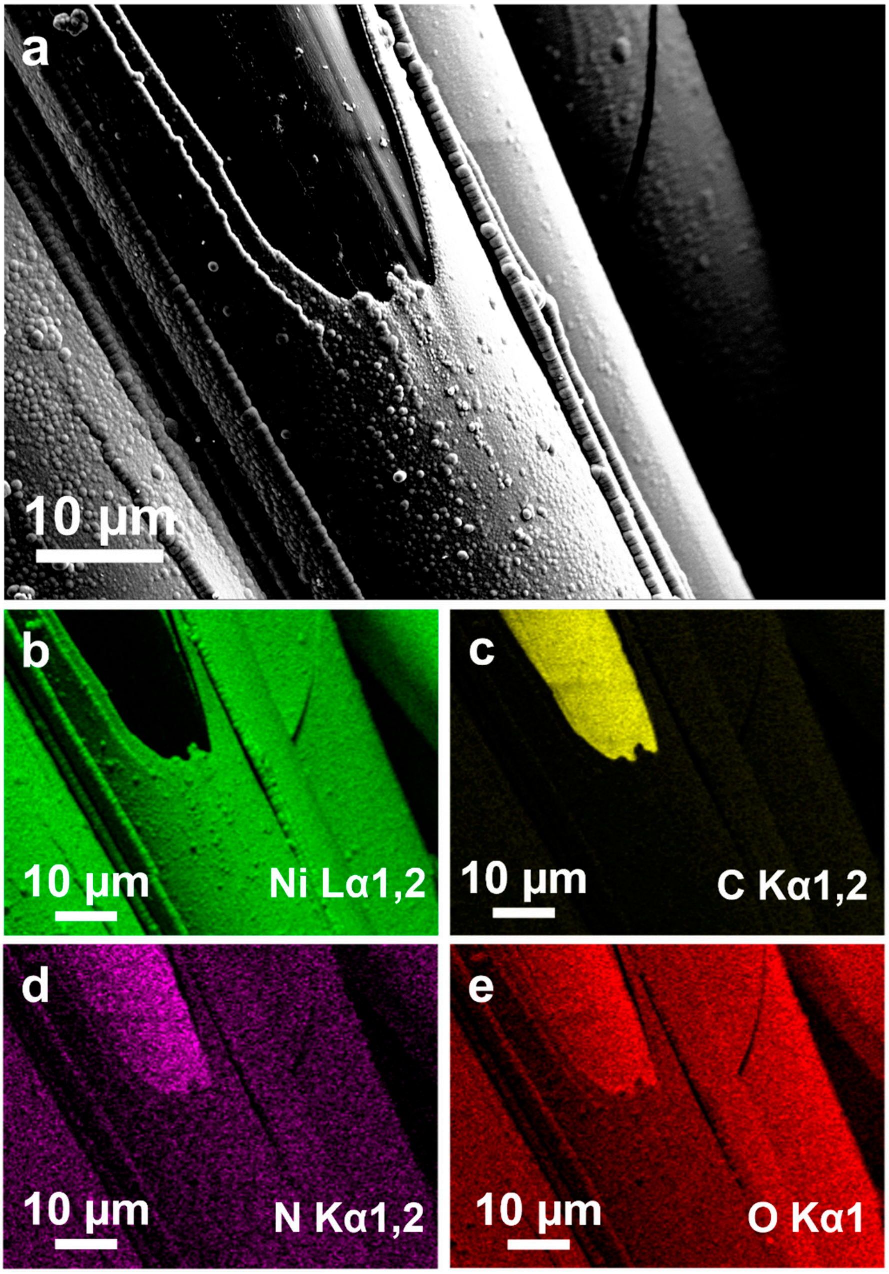

Figure S5. DMSO 1 wt.% modified ELD Ni-coated nylon yarns, (a) SEM image and (b-e) associated elemental mapping.

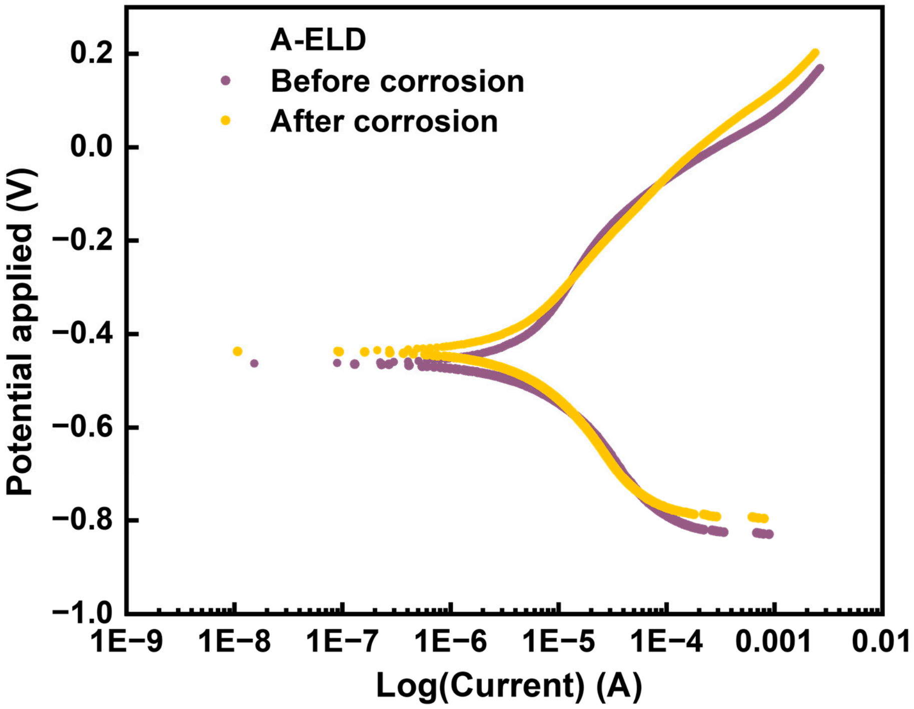

Figure S6. The potentiodynamic polarisation curves of A-ELD Ni-coated nylon yarn.
